# Interfacial Engineering of Ni–C/Ni–O–C Bonds in Carbon Nanotube Composites for High-Performance Non-Enzymatic Glucose Detection in Complex Beverage Matrices

**DOI:** 10.3390/molecules31101721

**Published:** 2026-05-19

**Authors:** Zhitao Yang, Xiaoben Yang, Meiwen Zhu, Ling Wu, Qianglin Li, Zheng-Hong Huang, Ming-Xi Wang

**Affiliations:** 1 School of Chemical and Environmental Engineering, Wuhan Institute of Technology, Wuhan 430205, China; yangzt1999@163.com (Z.Y.); 22509010054@stu.wit.edu.cn (X.Y.); 2 Chongqing Academy of Metrology and Quality Inspection, Chongqing 401123, China; zhumeiwen2007@126.com; 3 Hubei Province Key Laboratory of Coal Conversion and New Carbon Materials, School of Chemistry and Chemical Engineering, Wuhan University of Science and Technology, Wuhan 430081, China; wuling2018@wust.edu.cn; 4 Department of Material and Environmental Engineering, Chengdu Technological University, Chengdu 611730, China; 5 Key Laboratory of Advanced Materials Ministry of Education, School of Materials Science and Engineering, Tsinghua University, Beijing 100084, China; zhhuang@tsinghua.edu.cn

**Keywords:** composite electrode, Ni, carbon nanotube, enzyme-free glucose sensor, beverage analysis, interfacial engineering

## Abstract

The development of non-enzymatic glucose sensors for beverage analysis remains challenging due to insufficient active sites, poor conductivity, and limited stability in complex matrices. A nickel-carbon nanotube composite (Ni/CNT−600) was synthesized via in situ solvothermal deposition followed by pyrolysis at 600 °C under an inert atmosphere. The optimized Ni/CNT−600 featured uniform anchoring of Ni nanoparticles on CNTs through strong Ni–C and Ni–O–C interfacial bonds, validated by various characteristic techniques. The Ni/CNT−600 sensor exhibited exceptional sensitivity (538.48 μA mM^−1^ cm^−2^) and an ultralow detection limit (0.003 μM) in 0.1 M NaOH at +0.65 V, surpassing many reported metal-based and enzymatic sensors. It demonstrated remarkable selectivity against key interferents (e.g., ascorbic acid, uric acid). In real beverage samples (orange juice, grape juice, cola, green tea, milk), recovery rates ranged from 95.6% to 112.8%. This work demonstrates a well-defined Ni-CNT synergistic interface that contributes to enhanced non-enzymatic glucose sensing performance, effectively addressing matrix complexity in beverages.

## 1. Introduction

The accurate detection of glucose holds paramount importance across diverse fields, ranging from clinical diagnostics to food quality control [1,2]. In the beverage industry, monitoring glucose levels in fruit juices is crucial not only for assessing sweetness and authenticity but also for managing diabetic dietary intake and ensuring product consistency [3,4]. Conventional analytical techniques, such as chromatography and fluorescence spectroscopy, while offering high sensitivity, often suffer from limitations including complex sample pretreatment, bulky instrumentation, time-consuming procedures, and high operational costs, rendering them less suitable for on-site or rapid screening applications [5,6]. Among emerging alternatives, electrochemical sensing has garnered significant attention due to its inherent advantages, including high sensitivity, excellent selectivity, rapid response times, low cost, and potential for miniaturization into portable devices [7]. In recent years, various transition metal-based nanomaterials have been developed for non-enzymatic glucose sensing, showing great promise for applications in food and beverage analysis. For example, Wu et al. reported Co-doped NiMoO_4_ nanorods with oxygen vacancies prepared via microplasma and quenching, achieving high sensitivity and a low detection limit for glucose in food and serum samples [8]. Zhang et al. constructed a three-dimensional Co_X_P@NiCo-LDH heterogametes array on nickel foam, which exhibited an ultra-fast response and high sensitivity for glucose detection [9]. These studies, together with other recent reports, highlight the potential of interface-engineered bimetallic catalysts.

It is noteworthy that most high-performance non-enzymatic glucose sensors based on Ni or Co under alkaline conditions (typically 0.1 M NaOH), where the formation of active oxyhydroxide species (e.g., NiOOH, CoOOH) is facilitated [10]. As the reviews by Teymourian et al. and Hwang et al. have systematically discussed that the electrocatalytic oxidation of glucose on Ni- and Co-based electrodes proceeds via the redox cycling of M(OH)_2_/MOOH (M = Ni, Co) couples, which are only stable and active in basic solutions (typically pH > 12) [11,12]. Nevertheless, challenges remain in achieving stable and robust anchoring of active nanoparticles on conductive supports in complex beverage matrices, which motivated the present work. Although beverage labels typically declare sugar content, real-time glucose detection remains essential for quality control, detecting batch variations, and preventing label fraud [13]. Moreover, for diabetic individuals, accurate on-site glucose measurement in beverages is critical for dietary management, as label values may not always reflect actual concentrations due to degradation or inconsistent manufacturing processes [14]. These attributes make electrochemical methods particularly attractive for non-invasive and rapid analysis of glucose in complex matrices like fruit juices [15].

Electrochemical glucose sensing relies on catalytic materials that enable glucose oxidation at low potentials, offering a rapid and cost-effective approach [16]. Historically, enzyme-based electrodes, particularly those utilizing glucose oxidase (GOx), have dominated this field due to their high specificity and efficiency under physiological conditions [17,18,19]. Despite these advantages, enzyme-based sensors are plagued by intrinsic drawbacks such as operational instability from denaturation, stringent storage needs, and susceptibility to interferents in real samples [20,21]. These limitations have spurred considerable research into non-enzymatic alternatives, where nanomaterials with inherent catalytic activity provide enhanced durability and flexibility [22,23,24]. Among them, transition metal oxides/hydroxides and their composites stand out for their tunable electronic structures, abundance, and cost-effectiveness [25]. Nickel (Ni) emerges as a particularly compelling candidate, owing to the favorable redox properties of the Ni(OH)_2_/NiOOH couple and its ability to form active oxyhydroxide phases under alkaline conditions [26,27].

Despite the promising attributes of Ni-based catalysts, their practical application in non-enzymatic glucose sensing still faces persistent challenges. Firstly, the aggregation of Ni nanoparticles often leads to an insufficient number of active sites and diminished catalytic efficiency [28]. Secondly, the inherent poor electrical conductivity of pure nickel oxides, such as NiO, limits charge transfer kinetics, resulting in a rapid decay of sensor performance [29,30,31,32]. Furthermore, inadequate structural and electrochemical stability under complex matrix conditions and during long-term operation pose significant hurdles for their widespread adoption [33]. To address these issues, researchers have explored various strategies. For instance, nanostructure design (e.g., nanowires, nanosheets, porous structures) has been employed to increase surface area and expose more active sites [34,35,36]. Compositing Ni-based materials with highly conductive substrates (e.g., carbon materials, conductive polymers) can significantly enhance electrode conductivity and accelerate electron transport [37,38,39,40,41]. Moreover, the construction of heterostructures or the introduction of dopants has proven effective in optimizing the electronic structure and catalytic activity of Ni-based materials, while also improving their stability [31,42,43]. Nevertheless, achieving uniform dispersion of Ni nanostructures on conductive substrates, effectively suppressing aggregation, and simultaneously ensuring high catalytic activity, excellent conductivity, and robust structural integrity remain critical challenges for advancing non-enzymatic glucose sensor technology.

Carbon nanotubes (CNTs) have emerged as ideal substrate materials for constructing high-performance electrochemical sensors due to their unique properties. Their distinctive one-dimensional structure provides excellent electrical conductivity, facilitating efficient electron transfer between active sites and the electrode surface [33,36,43,44]. Beyond offering a high aspect ratio and structural support, the primary advantage of CNTs in this context lies in their ability to form highly conductive networks that not only enhance electron transfer kinetics but also provide numerous sites for the uniform dispersion of catalytic nanoparticles [44,45,46,47]. Additionally, CNTs possess remarkable mechanical strength and chemical stability, contributing to the overall robustness of the sensor. Decorating CNTs with catalytically active nanoparticles, such as nickel, can effectively combine the advantageous properties of both components, creating synergistic interfaces that significantly enhance catalytic performance and sensor longevity [46,48,49,50,51,52]. This synergistic effect manifests as CNTs serving as efficient electron transport channels while simultaneously providing stable anchoring sites for Ni nanoparticles, effectively inhibiting their aggregation and potentially optimizing the electronic structure of nickel through interfacial interactions, thereby further boosting the catalytic efficiency of glucose oxidation.

Therefore, to bridge the gap between the need for robust non-enzymatic glucose sensors and the limitations of existing Ni-based catalysts (e.g., insufficient active sites and poor stability in complex matrices), this work aims to develop a novel Ni-CNT composite through interfacial engineering of Ni-C and Ni-O-C bonds. By employing an in situ solvothermal deposition followed by controlled pyrolysis, we seek to achieve uniform anchoring of Ni nanoparticles on CNTs, which is expected to enhance electrical conductivity, catalytic activity, and structural stability. The optimized nanocomposite will be systematically evaluated for glucose detection not only in model solutions but also in real beverage samples (e.g., fruit juices and teas), thereby addressing the challenges of selectivity and reproducibility in complex environments. This approach demonstrates a synergistic interface design that could advance practical applications in beverage quality control and diabetic dietary management.

## 2. Results and Discussions

### 2.1. Synthesis and Characterization

**Scheme 1:**
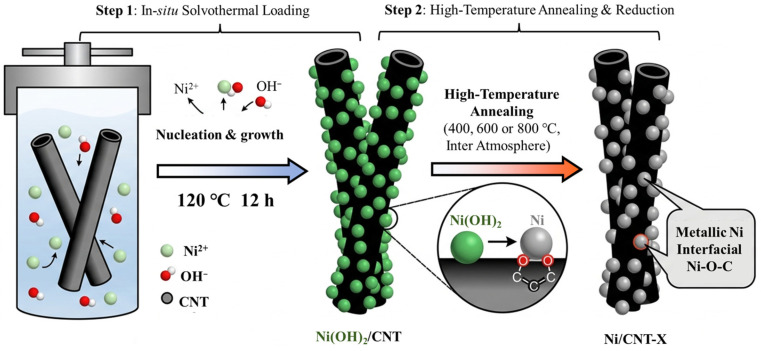
Schematic illustration of the synthesis of Ni/CNT−X composites via solvothermal deposition of Ni(OH)_2_ onto CNTs followed by high-temperature pyrolysis, forming Ni–C and Ni–O–C interfacial bonds.

Scheme 1 illustrates the synthesis principle of Ni/CNT−X composite materials, involving solvothermal in situ deposition of Ni(OH)_2_ followed by high-temperature annealing. During the solvothermal synthesis of Ni(OH)_2_/CNT composites, N, N-dimethylformamide (DMF) serves as an excellent dispersant for CNTs. It effectively disperses CNTs through non-covalent interactions, such as π−π stacking, thereby preventing their agglomeration and providing more active sites for the uniform loading of Ni(OH)_2_ onto the CNT surfaces [53]. Specifically, the attachment of Ni(OH)_2_ nanoparticles to CNTs occurs mainly through non-covalent interactions, including π−π stacking between the aromatic domains of CNTs and the Ni(OH)_2_ precursor, as well as hydrogen bonding between hydroxyl groups of Ni(OH)_2_ and oxygen-containing functional groups (e.g., –COOH, –OH) on the CNT surface [54]. DMF acts as a dispersant to prevent CNT agglomeration and promotes uniform nucleation [53]. Subsequent high-temperature pyrolysis converts these non-covalent interactions into strong Ni-C and Ni-O-C covalent bonds, ensuring robust anchoring and efficient electron transfer [44]. Concurrently, an aqueous sodium hydroxide (NaOH) solution is added to adjust the pH, ensuring a sufficient concentration of hydroxide nucleophiles to react with Ni^2+^, thus promoting the growth of Ni(OH)_2_ on the CNT surfaces [55]. The high-temperature annealing process is a crucial thermal reduction step, conducted under an oxygen-free (high-purity nitrogen) atmosphere. Its purpose is to reduce the Ni(OH)_2_ on the CNT surfaces to zero-valent nickel (Ni^0^) or to form stable Ni-O-C interfacial structures through dehydration [55]. This interfacial structure not only enables a more robust anchoring of nickel to the CNT surfaces but also enhances the overall stability and electrochemical performance of the composite material [56].

**Figure 1:**
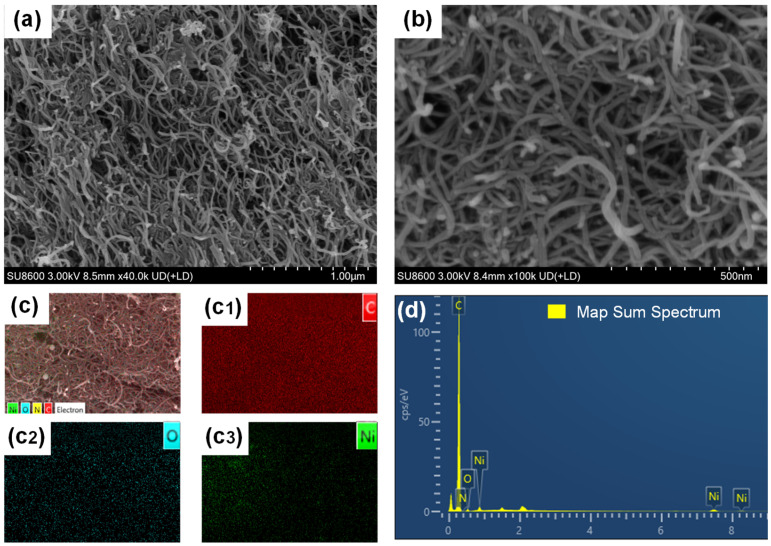
(**a**,**b**) FE-SEM, (**c**) EDS, and (**d**) EDS map sum spectrum of Ni/CNT−600.

Field-emission scanning electron microscopy (FE-SEM) images of raw CNTs and Ni/CNT−600 (Figure S1a and Figure 1a) reveal that the overall morphology remains largely unchanged after modification. The Ni/CNT−600 composite retains the characteristic irregular, curved tubular structure of CNTs, indicating that the synthesis method is mild and preserves the excellent interconnected network properties of the CNT substrate. Upon increasing the magnification to 100,000 times (Figure S1b and Figure 1b), it is evident that the surface of Ni/CNT−600 becomes significantly rougher, which confirms the successful surface functionalization [57]. To verify the successful loading of Ni onto the CNT surface, energy-dispersive X-ray spectroscopy (EDS) was employed for elemental characterization of the composite material. The homogeneous distribution of C, O, and Ni elements across the CNTs surface (Figure 1c) provides strong evidence for the successful and uniform in situ solvothermal deposition of nickel onto the CNTs. Furthermore, the semi-quantitative elemental mapping in Figure 1d indicates that the atomic percentage of Ni is approximately 0.4 at.%.

**Figure 2:**
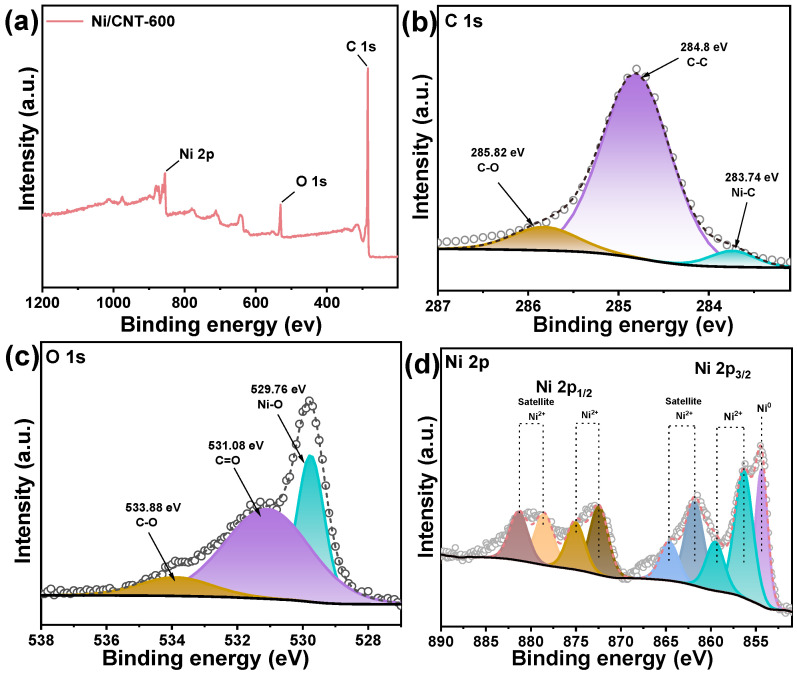
(**a**) XPS survey spectra; (**b**) high-resolution of C 1s, (**c**) O 1s and (**d**) Ni 2p spectra of Ni/CNT−600.

To elucidate the specific valence states and bonding configurations of Ni on the CNTs surface, X-ray photoelectron spectroscopy (XPS) analysis was performed on the Ni/CNT−600 [58,59,60,61]. The XPS survey spectrum (Figure 2a) clearly exhibits prominent peaks corresponding to C 1s, O 1s, and Ni 2p, with their relative atomic percentages detailed in Table S1. Notably, the relative atomic percentage of Ni is as high as 6.30 at.%, which is significantly greater than the values obtained from EDS. This discrepancy is attributed to the distinct probing depths of the two techniques: XPS is highly surface-sensitive, analyzing only the outermost layer (typically < 10 nm), whereas EDS offers a much deeper penetration depth (up to <1000 nm) [62,63]. Consequently, this finding strongly suggests that nickel is predominantly localized on the surface of the CNTs, as intended, which is crucial for maximizing the utilization efficiency of the active Ni species [64].

For a more in-depth analysis of the nickel valence states, high-resolution XPS spectra of C 1s, O 1s, and Ni 2p were deconvoluted. As depicted in Figure 2b, the C 1s spectrum can be resolved into three characteristic peaks centered at 283.74 eV (assigned to Ni–C), 284.8 eV (C–C), and 285.82 eV (C–O). The presence of the C–C peak confirms that the structural integrity of the carbon nanotubes is largely preserved during the material synthesis, thereby ensuring an efficient conductive network within the composite [65]. Furthermore, the C–O peak indicates the presence of a certain amount of oxygen-containing functional groups on the CNT surface [66]. These functional groups may originate from the inherent surface oxidation of the CNTs themselves, as well as from the dehydration of Ni(OH)_2_ precursors during synthesis [67]. Importantly, the peak at 283.74 eV, attributed to Ni–C bonding, provides compelling evidence for the direct chemical interaction between some Ni nanoparticles and the CNT substrate [68].

The O 1s high-resolution XPS spectrum, as shown in Figure 2c, can be deconvoluted into three distinct peaks at 529.76 eV (Ni–O), 531.08 eV (C=O), and 533.88 eV (C–O). The presence of the Ni–O species indicates either the formation of Ni-O-C bonds through the dehydration of Ni(OH)_2_ reacting with carbon, or the existence of a thin oxide layer on the surface of the Ni nanoparticles [69]. This Ni-O-C type bonding is highly beneficial for the robust anchoring of Ni onto the CNT surface. Moreover, a Ni/NiO core–shell structure or a partially oxidized surface state can positively influence electrocatalytic performance by promoting the interconversion of Ni^2+^/Ni oxygen reduction couples [70].

The Ni 2p high-resolution XPS spectrum (Figure 2d) exhibits complex multi-split features, comprising two main spin–orbit coupled peaks, Ni 2p3/2 and Ni 2p1/2, accompanied by their satellite peaks. Deconvolution of this spectrum reveals multiple characteristic peaks. The peak at approximately 854.1 eV is assigned to zero-valent Ni (Ni^0^), which corroborates the prior hypothesis regarding the presence of metallic nickel [71]. However, the predominant content of divalent Ni species suggests that nickel largely exists in the form of Ni-O-C or NiO within the composite [34]. The formation of Ni–C and Ni–O–C bonds identified by XPS analysis enables strong electronic coupling between Ni and CNT. DFT calculations on analogous Ni/CNT systems predict electron transfer from Ni to the CNT substrate [72], a directionality that is supported by the negative shift in the Ni 2p binding energy in Ni/CNT−600 relative to NiO references. Such electron redistribution optimizes the binding energy of glucose oxidation intermediates [73]. The functional importance of these interfacial bonds is further validated electrochemically: Ni/CNT−600 exhibits 1.7-fold higher electrochemically active surface area and significantly lower charge transfer resistance compared to Ni(OH)_2_/CNT and CNT (Figures S2 and S3), directly linking interfacial engineering to enhanced catalytic activity.

Comprehensive XPS analysis reveals that the Ni/CNT−600 composite successfully enriches and stably loads nickel onto the CNT surface. Its distinctiveness stems from [65,66]: (1) preserving the CNT framework, which underpins a conductive network; (2) forming strong Ni–C chemical bonds at the interface, enhancing stability; (3) featuring Ni-O-C interfacial bonds or a Ni/NiO core–shell structure, crucial for boosting electrochemical activity and stability; and (4) exhibiting coexisting metallic Ni^0^ and oxidized Ni^2+^ species, beneficial for diverse electrochemical reactions. Collectively, these structural and chemical bonding features endow Ni/CNT−600 with promising performance in glucose detection.

**Figure 3:**
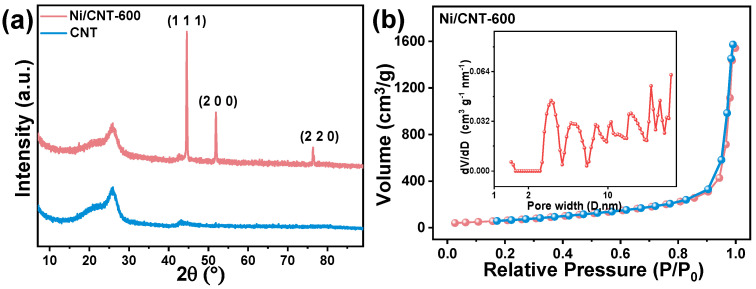
(**a**) XRD of Ni/CNT−600 and CNT; (**b**) N_2_ adsorption–desorption isotherms of Ni/CNT−600.

X-ray diffraction (XRD) patterns of the pristine carbon nanotubes and the Ni/CNT−600 composite (Figure 3a) both exhibit a broad diffraction peak at approximately 2θ = 25°, which is characteristic of the (002) crystallographic plane of graphitic carbon [44]. For the Ni/CNT−600 composite, a series of new, sharp diffraction peaks is observed. These new peaks, located at 2θ values of 44.4°, 51.9°, and 76.6°, correspond to the (111), (200), and (220) characteristic diffraction planes of metallic nickel (PDF#04-0850) [59], respectively. This result unequivocally demonstrates that through in situ hydrothermal loading followed by 600 °C pyrolysis treatment, nickel ions were successfully reduced to elemental metallic nickel and deposited onto the carbon nanotube surface. This finding corroborates the presence of zero-valent Ni, as previously inferred from the deconvolution results of the XPS Ni 2p spectrum [74].

Furthermore, the pore structure plays a crucial role in facilitating the transport of glucose molecules. Figure 3b displays the nitrogen adsorption–desorption isotherm of Ni/CNT−600, which presents a Type IV isotherm accompanied by a H3-type hysteresis loop. This indicates the existence of a mesoporous structure within the material [75]. The pore size distribution, derived from the Barrett-Joyner-Halenda (BJH) analysis (inset of Figure 3b), confirms that mesopores are dominant in Ni/CNT−600. Such a large pore structure is highly beneficial for the mass transfer of electrolyte molecules [76].

### 2.2. Electrochemical Performance of Ni/CNT−X/GCE for Glucose Oxidation

**Figure 4:**
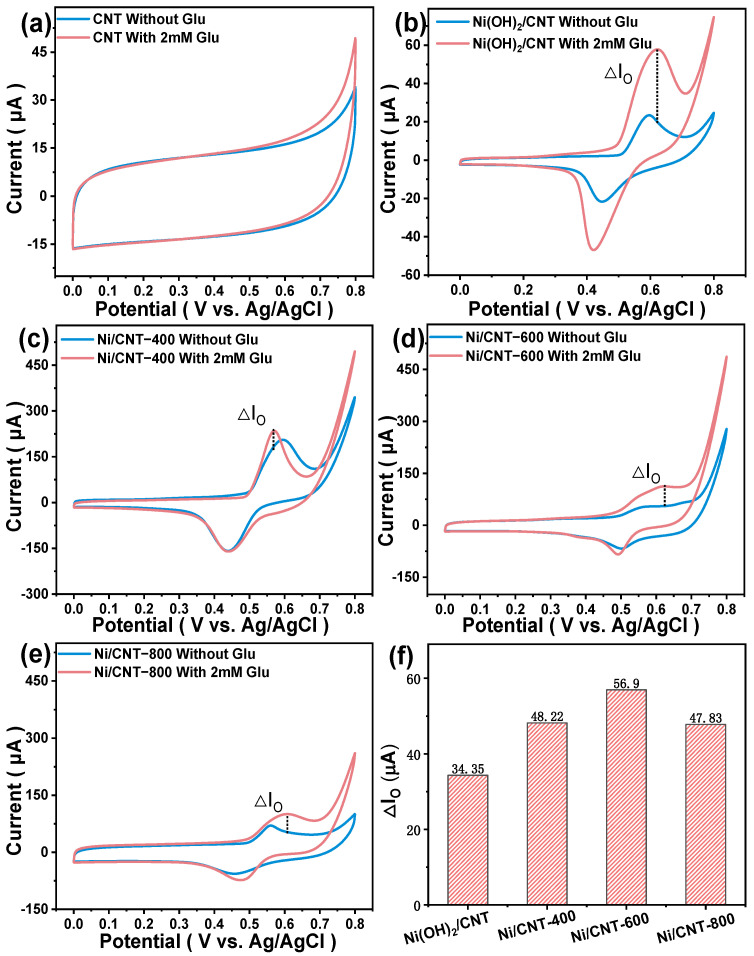
(**a**–**e**) CV curves of CNT, Ni(OH)_2_/CNT and Ni/CNT−X in 0.1 M NaOH without and with 2 mM glucose; (**f**) Comparison of current change values at glucose oxidation peak potential.

The glucose-sensing capabilities of all electrode materials were evaluated using a conventional three-electrode configuration, with various electrochemical tests conducted in a 0.1 M sodium hydroxide (NaOH) solution [77]. Figure 4a–e presents the cyclic voltammetry (CV) curves of CNT, Ni(OH)_2_/CNT and Ni/CNT−X (where X = 400, 600, or 800) in the presence (red lines) and absence (blue lines) of glucose. It is evident that the pure CNT shows negligible current change upon glucose addition, Ni(OH)_2_/CNT exhibits a modest increase, while the Ni-loaded CNT composites, synthesized at different pyrolysis temperatures, all exhibit an electrochemical response to glucose [78]. Specifically, upon the addition of glucose, a significant increase in current is observed within the potential range of 0.4–0.7 V. The peak current observed in the CV curves after glucose addition is defined as the glucose oxidation peak [79]. To facilitate a more direct comparison of the electrocatalytic glucose oxidation activity among the three materials, the current change ($∆I_{O}$) corresponding to the glucose oxidation peak potential was calculated and summarized in Figure 4f. Clearly, Ni/CNT−600 demonstrates the largest $∆I_{O}$, indicating its highest electrocatalytic activity for glucose oxidation [80]. It is noteworthy that although the absolute current of Ni/CNT−600 in the presence of glucose (red curve in Figure 4b) appears lower than that of Ni/CNT−400, the current change ($∆I_{O}$) upon glucose addition is the largest among all samples (Figure 4d). This is because Ni/CNT−400 exhibits a higher background current (blue curve) due to residual Ni(OH)_2_/NiO species, which contribute to non-specific oxidation. In contrast, Ni/CNT−600 possesses a more complete reduction to Ni^0^ and stronger Ni-C interfacial bonding, leading to lower background but higher catalytic efficiency for glucose oxidation. Therefore, ($∆I_{O}$) rather than the absolute current should be used to evaluate the electrocatalytic activity.

**Figure 5:**
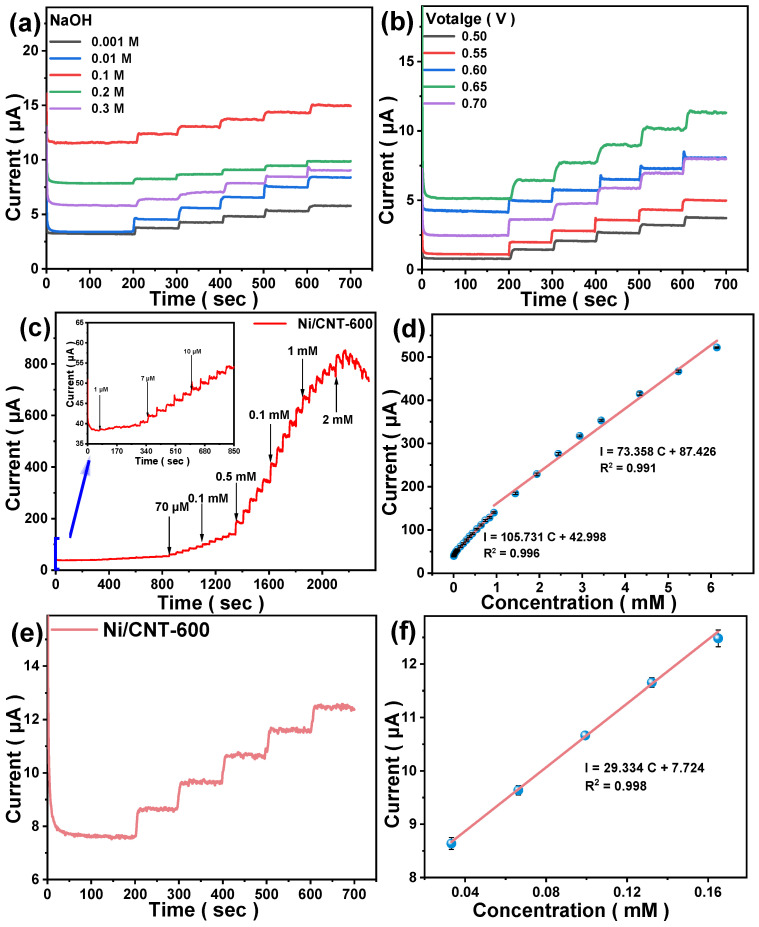
(**a**) i-t curves of Ni/CNT−600 in different concentrations NaOH (+0.65 V); (**b**) i-t curves of Ni/CNT−600 at different working potentials in 0.1 M NaOH solution; (**c**) amperometric responses of Ni/CNT−600/GCE upon stepwise injections of various glucose concentrations in 0.1 M NaOH at 0.65 V; (**d**) the linear correlation between glucose concentration and sensor’s response; (**e**) Amperometric response of Ni/CNT−600/GCE to trace glucose concentrations in 0.1 M NaOH at +0.65 V; (**f**) the linear correlation between glucose concentration and sensor’s response; Error bars represent standard deviation (SD) from three independent measurements.

To achieve optimal sensing performance for the Ni/CNT−600 electrode, the concentration of the alkaline solution (sodium hydroxide) and the applied potential were systematically investigated and optimized. Based on prior research, hydroxyl ions (OH^−^) play a crucial role in the electrocatalytic oxidation of glucose [81]. To determine the ideal sodium hydroxide concentration, the amperometric response of a Ni/CNT−600/GCE electrode was systematically evaluated in stirred sodium hydroxide solutions ranging from 0.001 to 0.3 M. As shown in Figure 5a, the current response generated by glucose addition was relatively weak at 0.001 M NaOH. While the response signal progressively intensified with increasing concentrations (0.01, 0.1, 0.2, and 0.3 M), a concomitant rise in background current was also observed under higher alkalinity conditions. This dual phenomenon might stem from the degradation of glucose into 5-hydroxymethylfurfural under strong alkaline conditions, thereby impeding the direct electrochemical oxidation process [82]. Among the concentrations tested, 0.1 M NaOH yielded the highest response current with a comparatively lower background current. Consequently, 0.1 M NaOH was selected as the testing condition for subsequent experiments.

Furthermore, the operating potential significantly influences the catalytic efficiency of sensing electrode materials. The current response of the Ni/CNT−600/GCE electrode at various operating potentials (0.5–0.7 V vs. Ag/AgCl) was investigated by successively adding 1 mM glucose to a 0.1 M NaOH solution (Figure 5b). The response signal was observed to continuously increase as the applied potential rose from 0.5 V to 0.65 V. At 0.7 V, the response current diminished due to the onset of oxygen evolution reactions. Therefore, considering the balance between signal strength and background noise, 0.65 V was ultimately chosen as the optimal potential for subsequent studies [83].

Chronamperometry (i-t) is a standard technique for evaluating sensor performance under constant applied potential. By recording the current response upon successive additions of glucose, key analytical parameters including sensitivity, linear range, and limit of detection (LOD) can be derived [84]. In a continuously stirred 0.1 M NaOH solution, at a constant potential of 0.65 V, varying concentrations of glucose standard solutions were successively introduced into the system every 50 s, and the corresponding current-time curves were recorded, as shown in Figure 5c. From the figure, it can be observed that after each glucose addition, the response current rapidly increased, reaching a new steady state within approximately 5 s. Figure 5d illustrates the correlation between the electrochemical signal (derived from i-t curves) and glucose concentration for the prepared modified electrode. All modified electrodes exhibited two distinct linear ranges in their current response versus glucose concentration profiles. This phenomenon suggests that the observed decline in sensitivity at higher concentrations arises from the accumulation of gluconolactone on the electrode surface, which hinders the diffusion of glucose to the active sites [85]. The two linear segments in Figure 5d do not intersect at exactly 0.94 mM, which is a typical phenomenon for non-enzymatic glucose sensors due to the adsorption of gluconolactone intermediates that partially block the electrode surface at higher glucose concentrations [85]. For practical quantification of glucose concentrations falling between 0.94 and 1 mM, either the calibration curve of the second linear range (0.94–6.14 mM) can be applied, or the sample can be diluted appropriately to bring the concentration within a single linear range. In this work, all beverage samples were diluted ten-fold before measurement, ensuring that the detected glucose concentrations fell well within the first linear range (0.004–0.94 mM). For the Ni/CNT−600/GCE electrode, the linear ranges were determined to be 0.004–0.94 mM and 0.94–6.14 mM, respectively, with corresponding regression equations provided:

(1) $$I_{1}\left(\mu A\right)=105.73\;C\left(mM\right)+42.99$$

(2) $$I_{2}\left(\mu A\right)=73.36\;C\;(mM)+87.43$$

The sensitivities, calculated by normalizing the slopes to the geometric electrode area, were 538.48 μA mM^−1^ cm^−2^ and 373.61 μA mM^−1^ cm^−2^, respectively. The limit of detection (LOD) was determined by measuring the amperometric response to successive additions of trace glucose concentrations (0.03–0.15 μM) in 0.1 M NaOH, as shown in Figure 5e, and was calculated using the formula LOD = 3σ/S, where σ is the standard deviation of the blank response and S is the slope of the calibration curve. A strong linear fit (R^2^ = 0.998) was found between the response current and concentration, and the LOD of the material was calculated using the formula “LOD = 3N/S” [85]. The Ni/CNT−600 electrode achieved a remarkably low LOD of 0.003 μM. Compared to some reported enzyme-free glucose sensors (as shown in Table 1), Ni/CNT−600 demonstrates high sensitivity, a low detection limit, and a broad linear range. All electrochemical measurements were repeated at least three times using independently prepared electrodes. The data are presented as mean ± standard deviation (SD). Error bars were added to all relevant figures (e.g., Figure 5d,f).

To gain a deeper understanding of the charge transfer kinetics, the electrochemically active surface area (ECSA) and charge transfer resistance (R_ct_) were further evaluated [89]. The double-layer capacitance (C_dl_), which is directly proportional to ECSA, was determined by cyclic voltammetry in the non-Faradaic region (vs. Ag/AgCl) at scan rates from 20 to 120 mV·s^−1^ (Figure S2). The C_dl_ values for CNT, Ni(OH)_2_/CNT, and Ni/CNT−600 were 0.701, 1.051, and 1.781 mF·cm^−2^, respectively. The significantly higher C_dl_ of Ni/CNT−600 (approximately 1.7 times that of Ni(OH)_2_/CNT and 2.5 times that of CNT) indicates a much larger number of accessible active sites. Electrochemical impedance spectroscopy (EIS) was performed in 0.1 M NaOH containing 2 mM glucose (Figure S3). The Nyquist plots showed that Ni/CNT−600 exhibited the smallest charge transfer resistance (R_ct_ = 38.68 Ω), compared to Ni(OH)_2_/CNT (42.74 Ω) and CNT (48.24 Ω). This confirms that the Ni–C and Ni–O–C interfacial bonds effectively facilitate electron transfer during glucose oxidation.

**Figure 6:**
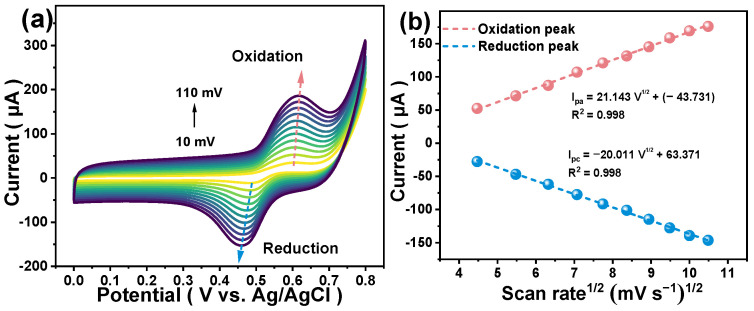
(**a**) CV curves of Ni/CNT−600 in 0.1 M NaOH + 2 mM Glucose solution at different scanning rates; (**b**) relationship between current and the square root of the scan rate.

A systematic study was also conducted using CV in a 0.1 M sodium hydroxide solution containing 2 mM glucose, at scan rates ranging from 10 to 110 mV/s. As illustrated in Figure 6a, both the anodic and cathodic peak currents increased with escalating scan rates, which indicates the strong ion and electron transfer capabilities of Ni/CNT−600, even at higher scanning speeds. Concurrently, a positive shift in the anodic potential was observed [28]. This phenomenon suggests that the glucose oxidation process occurring at the electrode surface is under kinetic control, as higher scan rates necessitate faster electrochemical reaction kinetics. Polarizing effects, leading to the observed peak potential shifts, typically occur when the electron transfer rate surpasses the diffusion rate of electrolyte species involved in the redox process [90]. To further investigate the electro-oxidation kinetics of glucose, plots of the peak currents (anodic peak current *I_pa_* and cathodic peak current *I_pc_*) versus the square root of the scan rate (v½) were constructed (Figure 6b). Both processes exhibited clear linear correlations, demonstrating:

(3) $$I_{pa}\left(\mu A\right)=\;21.143V^{1/2}-43.731$$

(4) $$I_{pc}\left(\mu A\right)=-20.011V^{1/2}+63.371$$

This linear dependency indicates that the electrocatalytic oxidation of glucose is diffusion-controlled, aligning with predictions from the chemical adsorption model [91].

**Figure 7:**
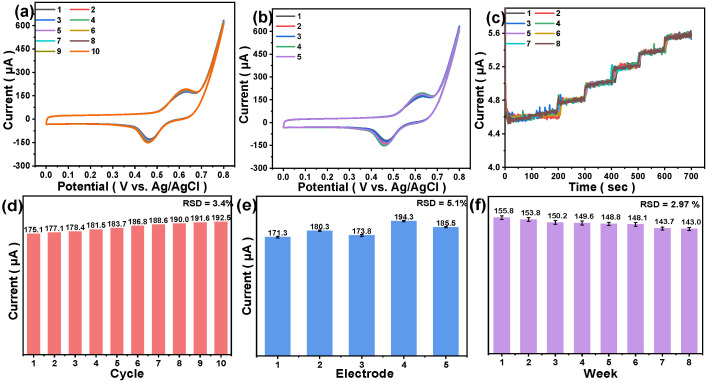
(**a**) CV curves of an identical electrode (Ni/CNT−600/GCE) running for 10 turns and (**b**) CV curves of assembled different electrodes (Ni/CNT−600/GCE) in 0.1 M NaOH + 2 mM Glucose solution; (**c**) amperometric responses of Ni/CNT−600/GCE to the same electrode tested weekly in 0.1 M NaOH solution; (**d**) histogram of the peak oxidation current according to (**a**); (**e**) histogram of the peak oxidation current according to (**b**); (**f**) weekly electrode current response value based on (**c**). Error bars represent standard deviation (SD) from three independent measurements.

To comprehensively evaluate the performance of the Ni/CNT−600/GCE sensor, the research team conducted rigorous tests on its reproducibility, repeatability, and long-term stability [66,92]. For the reproducibility assessment, a single Ni/CNT−600/GCE electrode was employed for 10 consecutive measurements of a 2 mM glucose solution under identical experimental conditions (as shown in Figure 7a), and the oxidation peak currents were recorded. The calculated relative standard deviation (RSD) for the oxidation peak currents was merely 3.4% (as depicted in Figure 7d), which strongly attests to the sensor’s excellent reproducibility and its capacity to deliver highly consistent measurement results under the same operating environment.

To further validate the sensor’s repeatability, five independent electrodes, each utilizing Ni/CNT−600 as the electrode material, were prepared using an identical synthesis method [66,92]. Subsequently, these electrodes were individually used to investigate their current responses to 2 mM glucose (as shown in Figure 7b). Analysis of the peak current detection results from these five independent electrodes yielded an RSD of 5.1% (as shown in Figure 7e). This outcome clearly indicates that sensors fabricated with Ni/CNT−600 material possess good batch-to-batch repeatability, which is crucial for scalable production and practical applications, as it ensures high comparability among different sensors.

Additionally, to thoroughly investigate the long-term stability of the Ni/CNT−600/GCE sensor, it was stored in a vacuum desiccator at 25 °C, and its oxidative current response to 2 mM glucose was periodically monitored (as shown in Figure 7c) [77]. After 8 weeks of storage (as depicted in Figure 7f), the sensing platform’s detection response to glucose showed only a minor decrease, retaining 91.8% of its initial response current, with a relative standard deviation of 2.97%. These data collectively confirm the sensor’s excellent long-term stability and robust durability under ambient storage conditions. The slight fluctuation observed in the signal was attributed by the researchers to the minor shedding of active Ni species, though this subtle change did not significantly compromise the sensor’s overall performance and reliability [93].

The reliability of electrochemical sensors in applications like complex biological analysis or beverage testing is often compromised by the interference from coexisting electroactive species and structurally analogous compounds [94]. This makes achieving high selectivity an essential requirement. In this study, i-t curve was used to test the anti-interference performance of the Ni/CNT−600/GCE in 0.1 M sodium hydroxide electrolyte at a constant potential of +0.65 V. A sequential addition protocol was employed, involving 1 mM glucose and 10 mM of common coexisting substances (ascorbic acid [AA], uric acid [UA], dopamine [DA], D-fructose, sucrose, lactose, NaCl, KCl), with experimental outcomes presented in Figure 4a, these interferents represent the major categories of electroactive biomolecules, sugars, and salts typically found in beverages. As shown, the Ni/TNC−600/GCE demonstrated excellent specific recognition capability for glucose. The current increments caused by interferents AA and DA were found to be approximately 1/3–1/5 of the glucose-induced response, whereas the response intensities of all other interferents were more than 10-fold lower than that of glucose. This excellent anti-interference characteristic makes it highly reliable for the detection of glucose in complex beverages and biological samples, which has an irreplaceable key value for the actual application scenarios.

The practical applicability of the Ni/CNT−600/GCE sensor was validated by quantifying glucose in real beverage samples, building on its established sensitivity, selectivity, and stability. The ultimate aim of developing high-performance non-enzymatic glucose sensors is reliable operation in complex, dynamic real-world settings, beyond idealized labs [95,96]. Assessing sensor performance amid coexisting interferents, pH shifts, and variable ionic strengths is therefore vital [97]. Five commercially available beverages (orange juice, grape juice, cola, green tea, milk) were selected as representative samples, given their inherently complex compositions: varying sugars, organic acids, proteins, and electroactive species. These matrices thus provide rigorous conditions to evaluate the sensor’s accuracy and interference tolerance [98].

**Figure 8:**
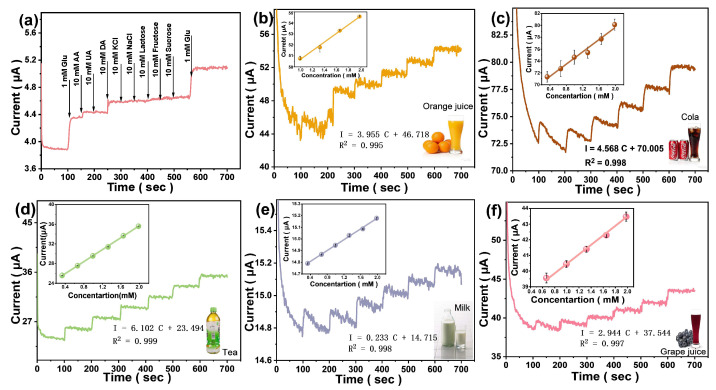
(**a**) Amperometric (i-t) responses of Ni/CNT−600 to successive additions of glucose (1 mM) and various interfering species (10 mM each) in 0.1 M NaOH at +0.65 V; Amperometric (i-t) responses of Ni/CNT-600 for glucose detection in various beverage samples: (**b**) orange juice, (**c**) cola, (**d**) green tea, (**e**) milk, (**f**) grape juice. Error bars represent standard deviation (SD) from three independent measurements.

To achieve precise quantitative analysis and effectively avoid matrix interference, the standard addition method was adopted as the core strategy [99,100,101]. In practice, each beverage sample was first diluted tenfold with 0.1 M NaOH solution. As shown in Figure 8b–f, upon successive glucose additions, the Ni/CNT−600/GCE exhibited rapid and well-defined current responses in all five beverage matrices, with stable current increments observed in the amperometric curves. The stepwise current responses were highly reproducible and closely resembled those obtained in pure 0.1 M NaOH electrolyte (Figure 5c), indicating that the complex matrix components did not significantly interfere with the electrocatalytic oxidation of glucose. In addition, as shown in Table S2, the recovery rate of glucose in all tested beverages (and at different concentration levels within each beverage) ranged from 96.5% to 116.2% (with only slightly lower recovery rates at low glucose concentrations in milk). These findings further confirm the robust anti-interference capability of the Ni/CNT−600/GCE sensor, which was previously demonstrated to effectively resist common interfering species such as ascorbic acid, uric acid, dopamine, and various sugars (Figure 8a).

## 3. Materials and Methods

### 3.1. Chemicals and Materials

Nickel chloride hexahydrate (NiCl_2_·6H_2_O), carbon nanotubes (CNTs), sodium hydroxide (NaOH), N, N-dimethylformamide (DMF), potassium ferricyanide (K_3_ [Fe(CN)_6_]), anhydrous ethanol, potassium chloride (KCl), D-anhydrous glucose, and Nafion (0.05 wt%) were all purchased from China National Pharmaceutical Group Chemical Reagent Co., Ltd. and used as received without further purification. Ascorbic acid (AA), uric acid (UA), dopamine (DA), lactose, D-fructose, and sucrose were of analytical grade and sourced from the same supplier. High-purity nitrogen (N_2_, 99.999%) was used for pyrolysis treatment. Deionized water (DI, 18.2 MΩ·cm) was prepared using a water purification system and employed throughout all experimental procedures. Commercial beverage samples (orange juice, grape juice, cola, green tea, milk) were purchased from a local supermarket and used for practical glucose detection without additional pretreatment except for dilution.

### 3.2. Synthesis of Ni/CNT−X

In a typical synthesis procedure, 0.6 g of NiCl_2_·6H_2_O and 0.3 g of CNTs were dispersed in 40 mL of DMF via ultrasonication. Subsequently, 20 mL of aqueous NaOH solution (0.1 M) was added dropwise to adjust the pH of the mixture to approximately 7.0 (neutral). After vigorous stirring, the resulting suspension was transferred into a 100 mL polytetrafluoroethylene (PTFE)-lined autoclave, sealed, and subjected to solvothermal treatment at 120 °C for 12 h to enable in situ deposition of Ni(OH)_2_ onto the CNT surface. After natural cooling to room temperature, the product was collected by centrifugation, washed three times with DMF, and dried under vacuum at 60 °C for 12 h to afford Ni(OH)_2_/CNT. The as-prepared Ni(OH)_2_/CNT was then thermally reduced under high-purity N_2_ atmosphere at a heating rate of 5 °C·min^−1^ to target temperatures of 400, 600, and 800 °C, respectively, and held at each temperature for 90 min. Upon cooling to room temperature, the resulting materials were denoted as Ni/CNT−X (X = 400, 600, 800). The pyrolysis temperatures were selected based on literature reports of the thermal behavior of Ni(OH)_2_ and CNT stability. It is established that Ni(OH)_2_ decomposes to NiO in the range of approximately 250–400 °C under an inert atmosphere [102]. Moreover, previous studies have shown that CNT surface oxygen groups, which facilitate Ni-C and Ni-O-C interfacial bond formation, decompose above 400 °C [72]. Accordingly, 400 °C was chosen as the lower boundary to ensure complete decomposition of the Ni(OH)_2_ precursor and to initiate Ni-CNT interaction; 600 °C was selected to achieve sufficient reduction in NiO to metallic Ni^0^ and to promote the formation of stable interfacial bonds; and 800 °C served as the upper boundary to evaluate possible degradation of CNTs or agglomeration of Ni nanoparticles, as excessively high temperatures may compromise CNT integrity [103].

### 3.3. Characterization

Field-emission scanning electron microscopy (FE-SEM, Hitachi SU8600, Tokyo, Japan) was used to observe the morphological features and nanostructure of the Ni/CNT−600 at an accelerating voltage of 3.0 kV. X-ray diffraction (XRD) patterns were recorded on a Bruker D8 Advance X-ray diffractometer with Cu Kα radiation (λ = 1.5406 Å) in the 2θ range of 5–80° at a scanning rate of 5 °C·min^−1^, for analyzing the crystal structure and phase composition. X-ray photoelectron spectroscopy (XPS, Thermo Scientific ESCALAB Xi+, East Grinstead, UK) with Al Kα radiation was utilized to characterize the surface chemical composition, elemental valence states, and chemical bonding environment of the materials. Nitrogen adsorption–desorption isotherms were measured at 77 K using an ASAP 2460 specific surface area and pore size analyzer, and the Brunauer–Emmett–Teller (BET) method was used to calculate the specific surface area, while the Barrett-Joyner-Halenda (BJH) model was applied to determine the pore size distribution and average pore diameter.

### 3.4. Fabrication of Modified Glassy Carbon Electrodes (GCE)

Prior to modification, the bare GCE (Φ = 5 mm) was pretreated to obtain a clean and smooth surface: the electrode was successively polished with 0.3 μm and 0.05 μm alumina (Al_2_O_3_) powder on a polishing cloth, and rinsed with DI water to remove surface abrasive residues. The polished GCE was then ultrasonically cleaned in DI water and anhydrous ethanol for 5 min each, and dried in an electric constant temperature incubator at 30 °C. The electrochemical activity of the pretreated GCE was verified by cyclic voltammetry (CV) in a 0.1 M KCl solution containing 5 mM K_3_ [Fe(CN)_6_]; the electrode was considered qualified for subsequent modification only if the potential difference between the oxidation and reduction peaks was less than 80 mV (indicating a reversible electrochemical process).

For the preparation of Ni/CNT−X modified GCE, 2 mg of Ni/CNT−X powder was accurately weighed and dispersed in 1 mL of anhydrous ethanol containing 0.05 wt% Nafion. The mixture was ultrasonically treated for 30 min to form a homogeneous and stable suspension. Subsequently, 10 μL of the above suspension was carefully dropped onto the pretreated GCE surface using a micropipette, and the modified electrode was dried in a 30 °C constant temperature incubator for 2 h to form a uniform and adherent sensing film. The modified electrodes were denoted as Ni/CNT−X/GCE for subsequent electrochemical measurements.

### 3.5. Electrochemical Characterization

All electrochemical characterizations and glucose sensing tests were implemented at room temperature in alkaline aqueous NaOH solutions on a CHI 760E electrochemical workstation (Shanghai Chenhua Instrument Co., Ltd., Shanghai, China), adopting a conventional three-electrode system: a Ni/CNT−X/GCE as the working electrode, a saturated calomel electrode (SCE) as the reference electrode, and a platinum (Pt) sheet as the counter electrode. Cyclic voltammetry (CV) was utilized to evaluate the electrocatalytic activity of Ni/CNT−X/GCE for glucose oxidation, with a potential window of 0–0.8 V and a scan rate of 50 mV·s^−1^; measurements were conducted in 0.1 M NaOH solution with and without 2 mM glucose for comparative analysis.

Chronoamperometry (i-t) with a fixed applied potential and a sampling time interval of 50 s was employed for the quantitative detection of glucose. Ni/CNT−X/GCE, the optimized material, was used to screen the optimal experimental conditions (NaOH concentration and applied potential) via i-t measurements in NaOH solutions of different concentrations (0.001, 0.01, 0.1, 0.2, 0.3 M) and at different applied potentials (0.50, 0.55, 0.60, 0.65, 0.70 V). Under the optimized conditions (0.1 M NaOH, 0.65 V), the i-t responses of Ni/CNT−X/GCE to successive additions of glucose with different concentrations were recorded to construct calibration curves, and the sensing sensitivity, linear range, and limit of detection (LOD) were further calculated. The LOD was determined according to the formula LOD = 3N/S, where N is the relative standard deviation (RSD) of the background current, and S is the slope of the linear calibration curve. The selectivity of Ni/CNT−X/GCE was evaluated by i-t measurements upon the addition of 1 mM glucose and 10 mM common interfering substances (ascorbic acid (AA), uric acid (UA), dopamine (DA), KCl, NaCl, lactose, D-fructose, sucrose) into a 0.1 M NaOH solution. The repeatability was tested by 10 consecutive CV scans of the same Ni/CNT−600/GCE in 0.1 M NaOH containing 2 mM glucose. The reproducibility was investigated by comparing the CV responses of five independently prepared Ni/CNT−600/GCE under the same test conditions. For ECSA evaluation, cyclic voltammetry was performed in the non-Faradaic region (vs. Ag/AgCl) in 0.1 M NaOH at different scan rates (Figure S2). The charging current density (ΔI) was calculated as (|I_a_| + |I_c_|)/(2A), where A = 0.19635 cm^2^ is the geometric area of the GCE. ΔI was then plotted against the scan rate (ν), and the slope of the linear fit gave the double-layer capacitance (C_dl_) [89]. EIS measurements were carried out at an applied potential equal to the open-circuit voltage (vs. Ag/AgCl) over a frequency range of 0.01 Hz to 100 kHz with an AC amplitude of 10 mV, in 0.1 M NaOH containing 2 mM glucose (Figure S3).

The long-term stability was assessed by measuring the i-t response of Ni/CNT−600/GCE to 2 mM glucose at weekly intervals for 8 weeks, with the electrode stored in air at room temperature when not in use. The response current was recorded each week, and the retention percentage was calculated relative to the initial response (week 1). This 8-week stability test is longer than the typical 2–4 week period reported in many non-enzymatic glucose sensor studies [35,42,46], further supporting the robust anchoring of Ni nanoparticles via Ni–C and Ni–O–C interfacial bonds.

### 3.6. Practical Glucose Detection in Beverage Samples

To verify the practical applicability of the optimized Ni/CNT−600/GCE sensor, glucose detection was carried out in real beverage samples (orange juice, grape juice, cola, green tea, milk). All beverage samples were first diluted 10-fold with 0.1 M NaOH solution (the supporting electrolyte) to simultaneously achieve the alkaline condition required for Ni-based glucose oxidation and to eliminate matrix effects. The dilution factor was accounted for in all recovery calculations, ensuring accurate quantification of original glucose concentrations. The standard addition method was adopted for quantitative analysis: a certain volume of the diluted beverage sample was added to the electrochemical cell containing 0.1 M NaOH, and the background current was recorded until stable. Then, glucose standard solutions with different known concentrations were successively added to the cell, and the corresponding i-t responses were recorded. The recovery rate was calculated according to the ratio of the detected glucose concentration to the spiked concentration, to evaluate the accuracy and reliability of the sensor for practical sample detection. All real-sample measurements were performed in triplicate, and the relative standard deviations (RSD) for recovery were below 5% for all beverages (Table S2). The sensor consistently performed well across different beverage matrices without signal drift, confirming its practical stability.

## 4. Conclusions

In this study, a novel Ni/CNT−600 nanocomposite was successfully synthesized through an in situ solvothermal approach followed by pyrolysis at 600 °C under an inert atmosphere. Comprehensive characterization via FE-SEM, XPS, and XRD analyses confirmed the uniform anchoring of Ni nanoparticles on carbon nanotubes via robust Ni-C and Ni-O-C interfacial bonds, which preserved the conductive CNT network while enhancing electrocatalytic activity. This interfacial engineering strategy directly addressed the key challenges highlighted in the introduction, such as insufficient active sites and poor stability in complex matrices, by providing abundant catalytic centers, efficient electron transfer, and structural integrity. Under optimized conditions (0.1 M NaOH, +0.65 V), the Ni/CNT−600-based sensor demonstrated exceptional performance for non-enzymatic glucose detection, with two linear ranges (0.004–0.94 mM and 0.94–6.14 mM), high sensitivities (538.48 and 373.61 μA mM^−1^ cm^−2^), and an ultralow detection limit of 0.003 μM. It exhibited remarkable selectivity against common interferents (e.g., ascorbic acid and uric acid), excellent reproducibility (RSD = 3.4%), repeatability (RSD = 5.1%), and long-term stability (91.8% retention after 8 weeks). Practical applicability was validated in real beverage samples (orange juice, grape juice, cola, green tea, and milk), achieving recovery rates of 96.5–116.2%, which underscores its reliability for complex matrix analysis. These findings present a rational interfacial engineering strategy for non-enzymatic glucose sensors, offering a robust solution for beverage quality control and on-site food analysis. We note that the sensor requires alkaline conditions (0.1 M NaOH) for optimal performance, which is a common limitation of Ni-based non-enzymatic sensors. Future work will focus on extending the sensor to neutral-pH operation through surface passivation or the design of alternative catalyst systems.

## Figures and Tables

**Table 1:** Comparison of Ni/CNT−600 with other enzyme-free glucose sensors.

Electrode	Sensitivity (μA mM^−1^ cm^−2^)	Linear Range (μM)	LOD (μM)	Ref.
Ni/NiO@C/GCE	9.73	0.5–2000	0.061	[35]
NiOOH/Ni(OH)_2_/MWCNT/GCE	3.17	0.0001–0.15	0.03	[44]
Ni-CNT-O/GCE	79.4	1–3000	0.5	[42]
Ag_5_-Cu_5_/MWCNTs/GCE	237.2	500–10,000	1.2	[78]
MXene@NiCo-LDH/CS6/GCE	154.05	1–3998	0.21	[86]
Pt1/Ni(OH)_2_/NG/GCE	220.75	10–2180	—	[87]
NiO/SiO_2_/NPOM/GCE	416	0–2000	0.84	[88]
Ni/CNT−600/GCE	538.48	4–940	0.003	This work
	373.61	940–6140		

## Data Availability

The data that support the findings of this study are available from the corresponding author, M.W., upon reasonable request.

## Supplementary Materials

The following supporting information can be downloaded at: https://www.mdpi.com/article/10.3390/molecules31101721/s1, Figure S1: SEM of CNTs; Figure S2: (a–c) Cyclic voltammograms of (a) CNT, (b) Ni(OH)_2_/CNT, and (c) Ni/CNT−600 in the non-Faradaic region (vs. Ag/AgCl) in 0.1 M NaOH at different scan rates o (d) Linear fitting of the charging current density (ΔI) versus scan rate (ν) for CNT, Ni(OH)_2_/CNT, and Ni/CNT−600.ΔI = |I_a_| + |I|/2A, where A = 0.19635 cm^2^ is the geometric area of the electrode; Figure S3: Nyquist plots of CNT, Ni(OH)_2_/CNT, and Ni/CNT−600 measured in 0.1 M NaOH containing 2 mM glucose at open-circuit voltage (vs. Ag/AgCl) over a frequency range of 0.01 Hz to 100 kHz with an AC amplitude of 10 mV; Table S1: XPS spectral data of Ni/CNT−600; Table S2: Standard addition test of actual sample glucose injection.
