# Supplementary material for: Interfacial Engineering of Ni–C/Ni–O–C Bonds in Carbon Nanotube Composites for High-Performance Non-Enzymatic Glucose Detection in Complex Beverage Matrices
Source: Molecules. 2026 May 19;31(10):1721. doi: 10.3390/molecules31101721 (PMC13209795; doi:10.3390/molecules31101721)
Supplement: Supplementary file 1 [file molecules-31-01721-s001.zip › molecules-4276586-supplementary.pdf]

Supplementary Information

Interfacial Engineering of Ni–C/Ni–O–C Bonds in Carbon Nanotube Composites for High-Performance Non-Enzymatic Glucose Detection in Complex Beverage Matrices

Zhitao Yang[1], Xiaoben Yang[1], Meiwen Zhu[2], Ling Wu[3], Qianglin Li[4,*], Zheng-Hong Huang[5], Ming-Xi Wang[1*]

[1] School of Chemical and Environmental Engineering, Wuhan Institute of Technology, Wuhan 430205, China.
[2] Chongqing Academy of Metrology and Quality Inspection, Chongqing 401123, China
[3] Hubei Province Key Laboratory of Coal Conversion and New Carbon Materials, School of Chemistry and Chemical Engineering, Wuhan University of Science and Technology, Wuhan 430081, China
[4] Department of Material and Environmental Engineering, Chengdu Technological University, Chengdu 611730, China
[5] School of Materials Science and Engineering, Key Laboratory of Advanced Materials Ministry of Education, Tsinghua University, Beijing 100084, China

* **Correspondence: lqlxp1010@163.com (Q.L.Li), wangmx14@wit.edu.cn (M.-X. Wang); Tel.: +86-27-87195680**

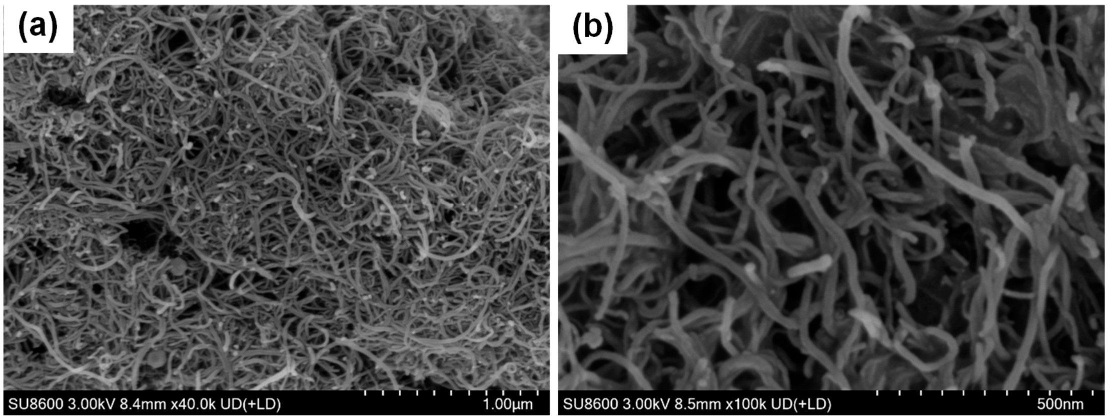

Figure S1 (a) SEM image of CNTs (b) high resolution SEM image of CNTs.

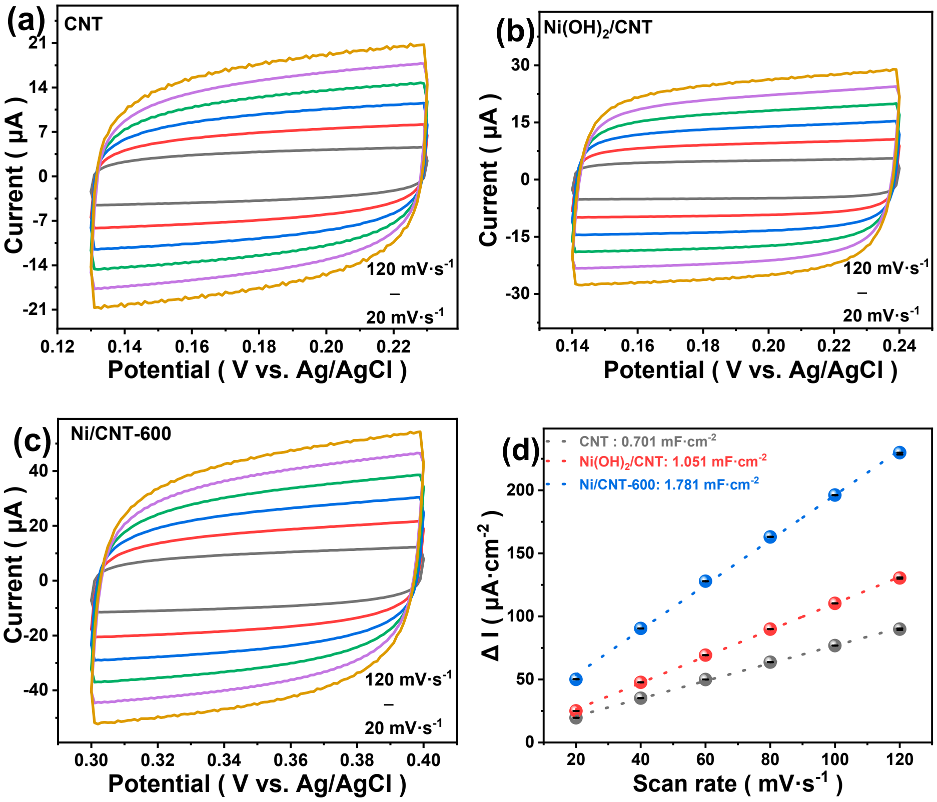

Figure **S2** (a–c) Cyclic voltammograms of (a) CNT, (b) $Ni(OH)_2$/CNT, and (c) Ni/CNT-600 in the non-Faradaic region (vs. Ag/AgCl) in 0.1 M NaOH at different scan rates o (d) Linear fitting of the charging current density (ΔI) versus scan rate (v) for CNT, $Ni(OH)_2$/CNT, and Ni/CNT-600.$\Delta I = |I_a| + |I$□$|/2A$, where A = 0.19635 cm² is the geometric area of the electrode.

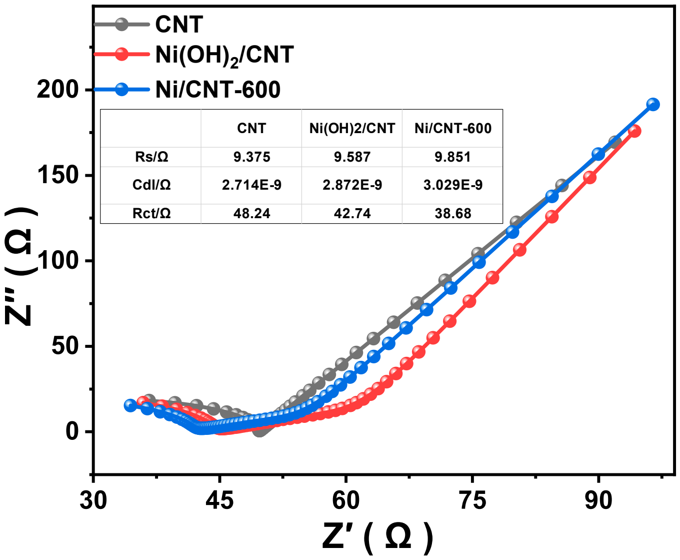

Figure **S3** Nyquist plots of CNT, $Ni(OH)_2$/CNT, and Ni/CNT-600 measured in 0.1 M NaOH containing 2 mM glucose at open-circuit voltage (vs. Ag/AgCl) over a frequency range of 0.01 Hz to 100 kHz with an AC amplitude of 10 mV.

Table S1 XPS spectral data of Ni/CNT-600

Element	Atomic %
C	85.43
O	8.27
Ni	6.30

Table S2 Standard addition test of actual sample glucose injection.

	**Orange juice**		**Cola**		**Green tea**		**Milk**		**Grape juice**	
Added	Founded	Recover	Founded	Recover	Founded	Recover	Founded	Recover	Founded	Recover
0.000	0.001		0.003		0.003		0.009		0.002	
0.332	0.333	99.9%	0.371	110.8%	0.341	101.7%	0.326	95.6%	0.377	112.8%
0.663	0.772	116.2%	0.644	96.8%	0.665	99.8%	0.485	71.8%	0.684	102.8%
0.993	1.020	102.5%	0.976	98.0%	0.991	99.5%	0.991	98.9%	0.986	99.0%
1.322	1.278	96.5%	1.287	97.1%	1.294	97.7%	1.356	101.9%	1.309	98.8%
1.650	1.660	100.5%	1.658	100.3%	1.665	100.7%	1.597	96.2%	1.612	97.5%
1.977	1.986	100.4%	2.004	101.2%	1.982	100.1%	1.987	100.1%	2.014	101.7%
